# Geriatrician-led multidisciplinary team management improving polypharmacy among older inpatients in China

**DOI:** 10.3389/fphar.2023.1167306

**Published:** 2023-05-04

**Authors:** Yi Song, Lihua Chen, Ying Liu, Xin Xia, Lisha Hou, Jinhui Wu, Li Cao, Li Mo

**Affiliations:** ^1^ The Center of Gerontology and Geriatrics, National Clinical Research Center of Geriatrics, West China Hospital, Sichuan University, Chengdu, Sichuan, China; ^2^ West China School of Nursing, Sichuan University, Chengdu, Sichuan, China

**Keywords:** polypharmacy, medication therapy management, multidisciplinary communication, aged, inpatients

## Abstract

**Background/Aim:** Polypharmacy is prevalent among older inpatients and associated with adverse outcomes. To determine whether a geriatrician-led multidisciplinary team (MDT) management mode could reduce medications use among older inpatients.

**Methods:** A retrospective cohort study was conducted in a geriatric department of a tertiary hospital in China with 369 older inpatients, including 190 patients received MDT management (MDT cohort), and 179 patients received usual treatment (non-MDT cohort). The primary outcome was to compare the changes of the amount of medications before and after hospitalization in two cohorts.

**Results:** We reported that MDT management significantly reduced the number of medications used in older inpatients at discharge (at home: *n* = 7 [IQR: 4, 11] vs at discharge: *n* = 6 [IQR: 4, 8], *p* < 0.05). Hospitalization with the MDT management had a significant effect on the change in the amount of medications (F = 7.813, partial-η^2^ = 0.011, *p* = 0.005). The discontinuance of medications was associated with polypharmacy at home (OR: 96.52 [95% CI: 12.53-743.48], *p* < 0.001), and the addition of medications was associated with a diagnosis of chronic obstructive pulmonary disease (COPD) (OR: 2.36 [95% CI: 1.02-5.49], *p* = 0.046).

**Conclusion:** The results indicated that the geriatrician-led MDT mode during hospitalization could reduce the number of medications used by older patients. The patients with polypharmacy were more likely to “deprescription” after MDT management, while the patients with COPD were more likely to be under-prescription at home, polypharmacy which could be made up for after MDT management.

## Introduction

The number of older adults has been continually growing around the world. With their extended lifespan, older adults are more likely to experience comorbidities and geriatric syndromes (American Geriatrics Society Expert Panel on the Care of Older Adults with Multimorbidity, 2012). The prevalence of older people aged ≥65 years old with comorbidities was 64.9% in Scotland (Barnett et al., 2012), which was 91.5% in the United States of America (Bahler et al., 2015). In China, 61.7%–86.3% of older adults suffer from different chronic diseases (Wang et al., 2021). They usually received long-term treatments with multiple medications (Marengoni et al., 2011).

Polypharmacy is most commonly defined in the literature as the concurrent use of five and more medications, including prescription drugs, over-the-counter (OTC) drugs, traditional Chinese medicine (TCM) and complementary medicines used by a patient (World Health Organization, 2022). Polypharmacy and potentially inappropriate medications (PIMs) are prevalent among older patients and are associated with adverse drug events (ADEs), drug interactions, hospitalizations, mortality and medical costs, especially in frail older patients (Mo et al., 2014; Poudel et al., 2016). Furthermore, inappropriate prescription not only refers to overprescribing but also includes misprescribing and underprescribing (San-José et al., 2014). Under such circumstances, older adults are frequently hospitalized due to acute exacerbated chronic diseases or accidents (Australian Institute of Health and Welfare, 2014). Previous studies found that the incidence of drug‒drug interactions in the older population was 28.1% (Weng et al., 2020), and 5%–10% of hospital admissions among older people were attributable to undesired side effects of drugs (Kratz and Diefenbacher, 2019).

The optimization of drug treatment is challenging, as both overtreatment and undertreatment issues may exist simultaneously in older people (Kaminaga et al., 2021). Therefore, some medication screening strategies have been created to help physicians optimize prescribing for older adults (Jansen et al., 2016). However, the impact of a single medication screening strategy may be limited, as it only addresses particular areas of the complex process of drug prescribing, such as providing drug names only, not reflecting the need for dose adjustments, interactions and so on (Pazan et al., 2019). Therefore, the objective of this study was to determine whether geriatrician-led MDT management could reduce the amount of medication used among older inpatients.

## Materials and methods

### Study design and sample size

This retrospective cohort study was conducted in the Geriatric Department of West China Hospital after approval of the Biomedical Ethics Sub-Committee of Sichuan University (2017–405) and was registered on the website of the Chinese Clinical Trial Registry (ChiCTR2000038003).

Older patients aged 65 years old and older admitted to the geriatric department from 1 September 2016, to 31 May 2017, were sampled for the study and were divided into the MDT cohort and the non-MDT cohort. We assumed that compared with the number of medications used at home, the average reduction in the number of medications used by older inpatients at discharge in the MDT cohort was about 1, with a total standard deviation of 3.4, assuming that the reduction in the number of medications use at discharge in the non-MDT cohort was 0 was an acceptable result, two independent sample T-tests (*α* = 0.05, 1-β = 0.8) were performed using PASS software to estimate the sample size, considering the sample shedding rate of 10%–20%, and finally concluded that at least 175 sample sizes were required for each cohort in this study.

**FIGURE 1:**
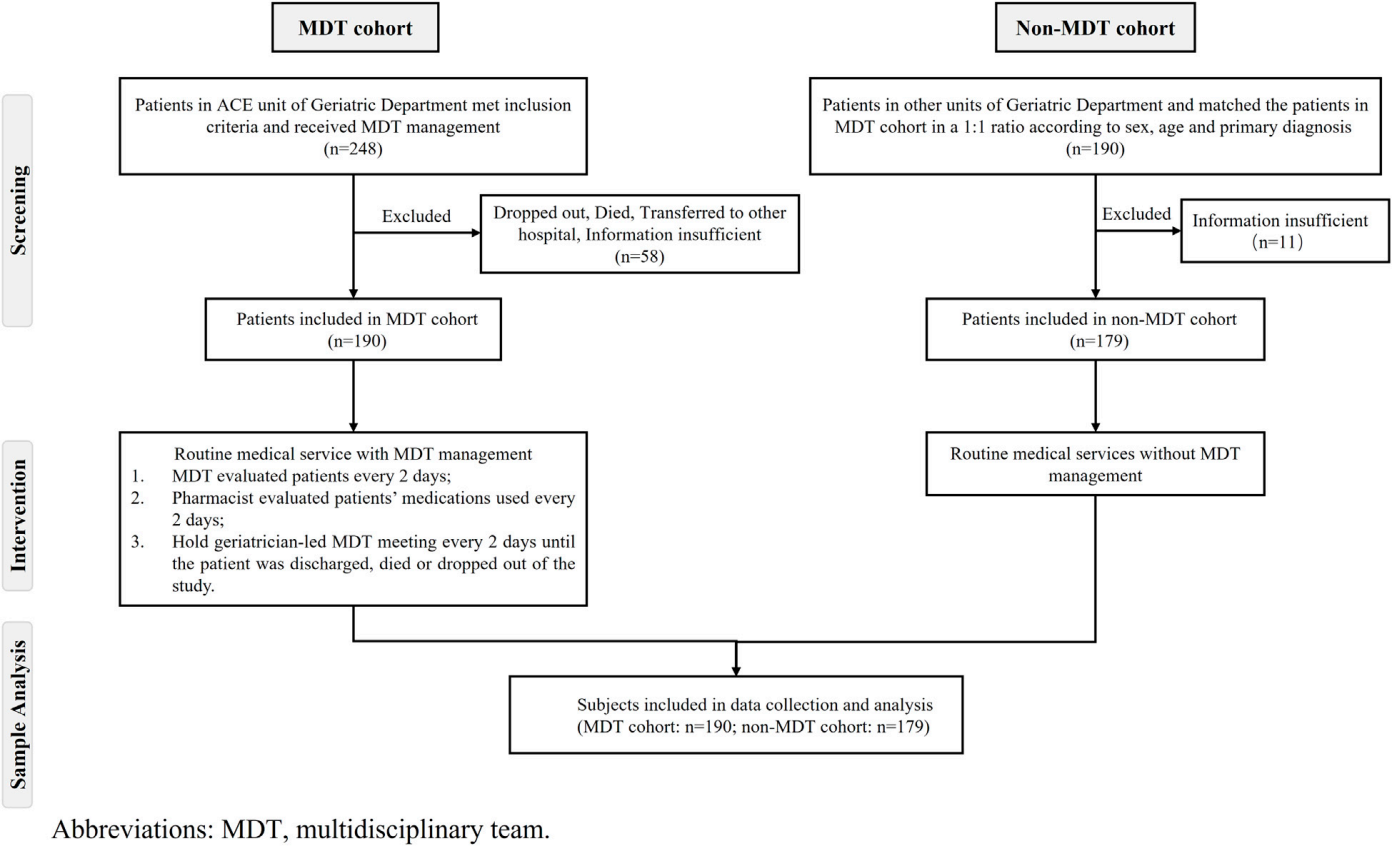
Flowchart of patient eligibility for the study.

To avoid physician-to-physician contamination, patients in the MDT cohort were recruited from the Acute Care for the Elderly Unit (ACE unit) of the Geriatrics Department, which was managed by the geriatrician-led MDT. Patients in the non-MDT cohort were recruited from other units of the Geriatrics Department except for the ACE unit, which was managed by the usual medical mode. Healthcare facilities, information insufficient and finally included 190 patients in the MDT cohort. Then, to achieve a 1:1 match with samples in the MDT cohort in terms of age, sex and primary diagnosis, STATA software were used for propensity score matching with a caliper of 0.01 to select samples in the non-MDT cohort. However, eleven patients in the non-MDT cohort were excluded due to incomplete data, so the final sample in the non-MDT cohort was 179 patients (Figure 1).

### Participants

Patients who met the following criteria were included in this study: 1) aged ≥65 years old and admitted to the Geriatrics Department of West China Hospital for an acute illness, and 2) agreed to receive comprehensive geriatric assessment and MDT management for patients in the MDT cohort. We excluded patients who 1) withdrew from MDT management for any reason, 2) were admitted to hospice care because of end-of-life care, 3) did not have a specific discharge plan, 4) lacked clear medication information, or 5) died or were transferred to another health institution without completing the entire management process.

### Management mode

Compared to the patients in the non-MDT cohort who received usual medical care, all the patients who matched the inclusion criteria in the MDT cohort received integrated medical management by a geriatrician-led multidisciplinary team. Each member in the MDT, including geriatricians, geriatric registered nurses, pharmacists, rehabilitation specialists, and nutritionists, would see the patients and evaluate related medical information on the admission day. In addition to these consultations, a geriatrician-led MDT meeting consisting of all the team members mentioned above was set up every 2 days, from day 2 of patient admission until discharge or withdrawal from the management. The goal of the meeting was to share patients’ information with all team members, provide a medication review, and discuss the comprehensive advanced care plan. All patients in the MDT cohort were evaluated every 2 days.

Specifically, the pharmacists evaluated and recorded patients’ medication use, including scheduled medications at home before admission, medication use and adjustment during hospitalization, and medication use on discharge. The pharmacists provided some patients’ information for medications to help geriatricians optimize prescribing, such as PIMs, potentially drug–drug interaction, potentially drug–disease interaction, wrong drug dosage, wrong frequency or route of administration, repeated medication use, possibly missed medication, unreasonable medication duration.

### Data collection

We collected data on patient demographics, including age, sex, medical record number, diagnostic diseases, date of admission and discharge, total hospitalization costs and medication costs, from the electronic medical records in West China Hospital. We also collected information on the medication profile from the electronic medical records, mediation administration records and MDT evaluation record sheets (for the MDT cohort) 1) at home before admission, 2) during hospitalization, and 3) at discharge. Polypharmacy was defined as the concurrent use of five and more medications, including OTC drugs, prescription and/or traditional and complementary medicines used by a patient.^6^

### Statistical analysis

The baseline characteristics of the patients were described with descriptive statistics, and the data were summarized as the median (interquartile range [IQR]) or number (percentage) according to the distribution of the variables. The changes in the number of medications used before and after hospitalization between the MDT cohort and the non-MDT cohort were compared by the Mann-Whiney *U* test. The amount of medications used among the time points of at home, during hospitalization, and at discharge in each group were compared by the Friedman test. The 2 × 2 factorial design was conducted to verify the effect of the MDT mode on the amount of medication used by participants. To determine the relative factors associated with the discontinuation or addition of medications after MDT management, a multivariate analysis for the factors associated with a change in medication before and after hospitalization was conducted by binary logistic regression analysis (represented by estimating odds ratios [OR] and 95% confidence intervals [CI]). *p* < 0.05 was considered statistically significant. Statistical analysis was performed with SPSS 26.0 (IBM Corp, Chicago, IL, U.S. A) and STATA 15.0 (STATA Corp, College Station, TX).

## Results

### Patient characteristics

In total, 248 inpatients aged 65 years old and above received MDT management from 1 September 2016 to 31 May 2017. A total of 190 patients (male: n = 140, 74%) aged 86 years old (IQR: 82, 89) were included in the MDT cohort. Meanwhile, the non-MDT cohort included 190 patients who matched the MDT cohort 1:1 in age, sex and primary diagnosis. Finally, a total of 179 patients (male: n = 135, 75%) aged 85 years old (IQR: 80, 89) were included in the non-MDT cohort (Table 1). The prevalence of comorbidities among older inpatients is shown in Table 2.

**TABLE 1:** Comparison of older inpatients’ characteristics and medication change between the MDT cohort and the non-MDT cohort.

	Non-MDT cohort	MDT cohort	*P*
	(n = 179)	(n = 190)	
Sex			
Male (n, %)	135 (75)	140 (74)	0.702
Female (n, %)	44 (25)	50 (26)	
Age (years)	85 (80.89)	86 (82.89)	0.495
Length of stay (days)	19 (12.28)	14 (10.19)	<0.05
HE ($)	2,723 (1755,4,717)	2,628 (1916,3,833)	0.595
ME ($)	701 (385,1367)	776 (496,1179)	0.435
ME/HE (%)	26 (18.37)	29 (21.36)	0.068
Diagnostic diseases n)	9 (7.13)	8 (6.11)	0.001
Number of patients with Polypharmacy (n, %)			
At home	114 (64)	137 (72)	0.083
During hospitalization	165 (92)	186 (98)	0.011
At discharge	139 (78)*	131 (69)	0.059
Number of Medication used			
AT home	6 (3.1)	7 (4.11)	0.362
During hospitalization	12 (9.17)	13 (9.17)	0.508
At discharge	7 (5.10)	6 (4.8)**	<0.05
Number of medications changes***	0 (-2.3)	-1 (-4.2)	0.001
Number of patients with medication changes at discharge (n, %)			
Increase	151 (84)	163 (86)	0.699
Decrease	140 (78)	167 (88)	0.013

Abbreviations: HE, Hospitalization expenditure; ME, medicine expenditure.

Data are the median (interquartile range) or number (percentage) unless indicated.

*: *p* < 0.001, compared with the number of patients with polypharmacy at home in the same cohort.

**: *p* < 0.05, compared with the number of medications used at home in the same cohort.

***The difference between the number of medications used by patients at the time of discharge and at home.

**TABLE 2:** Top ten diagnostic diseases in the MDT cohort and non-MDT cohort.

Non-MDT cohort (n = 179)		MDT cohort (n = 190)	
Diagnostic disease	Number of patients (n, %)	Diagnostic disease	Number of patients (n, %)
Hypertension	121 (68)	Pneumonia*	129 (68)
ICVD	104 (58)	COPD*	123 (65)
COPD	90 (50)	Hypertension	119 (63)
BPH	77 (43)	BPH	73 (38)
CHD	74 (41)	CHD	70 (37)
AS	74 (41)	Cardiac insufficiency	60 (32)
Pneumonia	70 (39)	ICVD*	59 (31)
DM	63 (35)	DM	49 (26)
Arrhythmia	47 (26)	Arrhythmia	47 (25)
Cardiac insufficiency	45 (25)	Mental disorder	42 (22)

Abbreviations: ICVD, ischemic cerebrovascular disease; COPD, chronic obstructive pulmonary disease; BPH, benign prostate hyperplasia; CHD, coronary heart disease; AS, atherosclerosis; DM, diabetes mellitus.

*: *p* < 0.05, Compared with the non-MDT, cohort, the prevalence of patients with the diagnostic disease was significantly different.

### Geriatrician-led MDT management shortened the hospital stays of older inpatients

Although there were no significant differences between the total costs of hospitalization or medication costs between the two groups, the MDT mode significantly shortened the length of hospital stay of patients compared to the non-MDT cohort (MDT cohort: n = 14 days [IQR: 10, 19] vs non-MDT cohort: n = 19 days [IQR: 12, 28], *p* < 0.05) (Table 1).

### The MDT management mode improved medication profile changes among older inpatients

In this study, 68.02% (*n* = 251) of older patients had polypharmacy at home, and there was no significant difference between the MDT cohort and the non-MDT cohort. During hospitalization, the prevalence of polypharmacy increased both in the MDT cohort and the non-MDT cohort. Although 139 patients (78%) in the non-MDT cohort were still prescribed five and more medications at discharge (compared to at home: *n* = 114 (64%), *p* < 0.001), the number of patients with polypharmacy in the MDT cohort did not change significantly at discharge (at discharge: n = 131 (69%) vs at home: *n* = 137 (72%), *p* > 0.05) (Table 1).

Interestingly, we found that the geriatrician-led MDT mode reduced the amount of medications used by one in older patients at discharge compared to the amount of medications used at home (at home: *n* = 7 [IQR: 4, 11] vs at discharge: *n* = 6 [IQR: 4, 8], *p* < 0.001). In contrast, the number of medications used at discharge did not decrease among patients in the non-MDT cohort (at home: *n* = 6 [IQR: 3, 11] vs at discharge: *n* = 7 [IQR: 5, 10], *p* > 0.05) (Table 1).

Overall, approximately 85% of older patients added new medications, and more than 78% of older patients stopped some medications used at home upon discharge in both cohorts (Table 1). Some medications used at home were discontinued 1,360 times upon discharge, of which approximately 60% (*n* = 811) occurred in the MDT cohort. Meanwhile, the frequency of new medications added at discharge was 1,177 times, and there was almost no difference between the two cohorts (MDT cohort: *n* = 574 (49%) vs non-MDT cohort: *n* = 606 (51%), *p* > 0.05) (Table 3).

**TABLE 3:** Top five types of medications added/stopped at discharge in the MDT cohort and non-MDT cohort compared with at home.

	Frequency of stopped medications (n=1360), n (%)		Frequency of added medications (n=1177), n (%)	
MDT cohort	Total frequency	811 (100)	Total frequency	574 (100)
	TCM for others ^a^ *	98 (12)	OTC drugs	38 (7)
	TCM for CCD ^b^ *	90 (11)	GC	37 (6)
	OTC drugs	86 (11)	β receptor agonists ^c^	32 (6)
	Antiplatelet drugs	32 (4)	Mucus relief agents	26 (5)
	ACEI/ARB	31 (4)	Montelukast	25 (4)
non-MDT cohort	Total frequency	549 (100)	Total frequency	606 (100)
	OTC drugs	49 (9)	OTC drugs	39 (6)
	TCM for others	46 (8)	Statins	36 (6)
	TCM for CCD	31 (6)	Antiplatelet drugs	32 (5)
	Externally applied agent	30 (5)	Mucus relief agents	27 (4)
	β receptor agonists ^c^	15 (3)	ACEI/ARB	20 (3)
	Mucus relief agents	15 (3)	PPI	20 (3)
			Externally applied agent	20 (3)

Abbreviations: TCM, traditional Chinese medicine; ACEI/ARB, angiotensin-converting enzyme inhibitor/angiotensin receptor blocker; OTC, drugs, overthe-counter drugs; PPI, proton-pump inhibitor; GC, glucocorticoids.

*TCM, for others; TCM, for non-cardiovascular and non-cerebrovascular disease.

**TCM, for CCD: TCM, for cardiovascular and cerebrovascular disease.

***β receptor agonists for respiratory disease.

****p < 0.05, Compared with the non-MDT, cohort, the amount of medication stopped or added at discharge was significantly different.

Compared to at home, approximately 10% of medications discontinued at discharge belonged to OTC drugs both in the MDT cohort and the non-MDT cohort. Twelve percent (*n* = 98) of medications stopped at discharge in the MDT cohort, and 8% (*n* = 46) in the non-MDT cohort were TCM for non-cardiovascular and non-cerebrovascular disease treatment (*p* < 0.05). In the MDT cohort, TCMs for cardiovascular and cerebrovascular disease treatment were discontinued 90 times (11%) at discharge, while only 31 times (6%) were discontinued in the non-MDT cohort (*p* < 0.05). The medicine category with the most added frequency at discharge was OTC drugs in both cohorts. Other drugs stopped or added at discharge are shown in Table 3.

### Related factors associated with the discontinuation or addition of medication in older patients

This study illuminated that hospitalization with the MDT management mode had a significant effect on the change in the number of medications at the time of discharge and at home in older inpatients (F = 7.813, partial-η^2^ = 0.011, *p* = 0.005), while routine hospitalization without the MDT management mode had no significant effect on it (F = 0.431, partial-η^2^ = 0.001, *p* = 0.512).

Our study intended to explore the factors related to discontinuation or addition of medications in older patients after MDT management and incorporated patients’ age, gender, the number of comorbidities, polypharmacy at home and types of diseases into the analysis of related factors. The binary logistic regression model showed that the probability of medication discontinuation at discharge was associated with polypharmacy at home (OR: 96.52 [95% CI: 12.53–743.48], *p* < 0.001), and the probability of medication addition was associated with COPD (OR: 2.36 [95% CI: 1.02–5.49], *p* = 0.046) after adjusting for patient age, sex, and the number of comorbidities (Table 4).

**TABLE 4:** Factors associated with the discontinuation or addition of medication at discharge

	Medication discontinuation				
	Chi-square test		Binary logistic regression		
	*χ2*	*P*	OR	95%CI	*P*
Polypharmacy	59.730	<0.001	96.516	12.53-743.48	<0.001
More than 8 coexisting diseases	3.390	0.066	-	-	0.428
Hypertension	4.102	0.043	-	-	0.092
CHD	4.255	0.039	-	-	0.692
COPD	3.278	0.070	-	-	0.624

	Medication addition				
	Chi-square test		Multivariate analysis		
	*χ2*	*P*	OR	95%CI	*P*
COPD	3.794	0.051	2.36	1.02-5.49	0.046
More than 8 coexisting diseases	2.869	0.090	-	-	0.307
Cardiac insufficiency	4.094	0.043	-	-	0.245
Arrhythmia	7.504	0.022	-	-	0.105
BPH	3.491	0.062	-	-	0.129
Mental disorder	4.626	0.094	-	-	0.074

Abbreviations: OR, odds ratio; CI, confidence interval; COPD, chronic obstructive pulmonary disease; CHD, coronary heart disease; BPH, benign prostatic hyperplasia.

Factors with *p* < 0.1 in the chi-square test were included in the binary logistic regression analysis.

## Discussion

### Geriatrician-led MDT management optimizing prescription for older inpatients

Polypharmacy and prescription omission often coexist in older adults. Polypharmacy was common among older adults, and the prevalence increased with age (Page et al., 2019; Pazan and Wehling, 2021). Our study demonstrated that 68.02% of older patients had polypharmacy at home, which worsened after hospitalization. Polypharmacy is well known to be associated with an increased risk of PIMs and adverse drug reactions (ADRs). It was also an important risk factor that resulted in 90% of older adults being hospitalized due to ADRs (Pedros et al., 2016). Other adverse health outcomes, such as subsequent fractures, acute renal failure, disability, physical and cognitive function impairment, readmissions and mortality, were also significantly associated with polypharmacy (Gómez et al., 2014; Wastesson et al., 2018).

Our study found that the effect of a geriatrician-led MDT in prescription was not only to reduce the number of medications used by older patients but also to optimize the overall medication plans. Although there was not a reduction in the number of patients with polypharmacy at discharge, geriatrician-led MDT management reduced the number of medications by one in older patients at discharge compared to the number of medications used at home. The World Health Organization (WHO) estimated that approximately 30%–50% of patients could not take medications as prescribed by doctors (Ulley et al., 2019), and older patients who suffered from multiple chronic diseases, hypofunction, cognitive and sensory disorders often had poor adherence to complex and excessive drugs for a long time (de Araujo et al., 2020). Thus, avoiding polypharmacy and optimizing prescribing are increasing challenges in clinical practice, and effective interventions to further improve drug therapy in older adults are strongly recommended.

Our study showed that a geriatrician-led MDT mode was more effective in reducing unnecessary TCM prescribing than conventional medical services. It is well known that TCMs are widely used in China and other East Asian countries and are increasingly used in Europe (Wang et al., 2018). Although most TCMs lack clear indications and clinical benefits, they are still an important part of long-term family medication regimens (Lau et al., 2001; Xiong et al., 2015). Due to cultural causes and national regulations, it is easy to obtain TCMs in China without a physician’s prescription. However, the interactions between components of TCMs may lead to potential ADRs, such as drug-induced liver injury, serotonin syndrome, renal impairment, rhabdomyolysis, and acute delirium (Wang et al., 2018).

Furthermore, our study proved that a geriatrician-led MDT mode could significantly reduce the inappropriate use of OTC drugs in older patients. OTC drugs usually contain prescription drug ingredients with an active effect. Taking OTC drugs and prescription medications at the same time may lead to repeated medications and increase the potential risk of overdose (Yang et al., 2021). In China, OTC drugs accounted for 12.6% of ADEs, among which people over 60 years old accounted for 24.4% (Yang et al., 2021). However, only 45% of Chinese residents had a certain understanding of OTC drugs (Yang et al., 2021). In China, clinical pharmacists and doctors rarely intervened in the use of OTC drugs in older adults during non-hospitalization.

### Geriatrician-led MDT management improves prescription omissions for older patients with COPD

In this study, older patients with COPD who added new medications at discharge were 2.4 times more likely than older patients without COPD after MDT management, suggesting that the patients with COPD were more likely to be at risk of inadequate medication use at home.

It has been proven that more than 80% of patients with COPD suffer from one or more other chronic diseases (Divo and Celli, 2020). Therefore, patients with COPD were more likely to experience polypharmacy (Hanlon et al., 2018). Many studies have shown that medication adherence rates in patients with COPD were only 10%–40% (Abdulsalim et al., 2018). Approximately 15% of patients with respiratory diseases were unwilling to accept new prescription drugs and stopped taking medications that controlled respiratory symptoms after approximately 6 months (Abdulsalim et al., 2018). Thus, patients were vulnerable to potential prescription omissions (PPOs) during the non-acute onset of COPD. Our study also showed that the management mode of MDT could effectively optimize long-term drug therapy in patients with COPD and reduce PPOs.

Drug prescription is a complex process for older adults with comorbidities. Relying on the geriatrician alone, it is difficult to fully grasp the patient’s information of medications, illness, functional status, medication adherence and other issues. The geriatrician-led MDT management mode is a novel clinical practice to improve quality of life for older patients. Our study also illuminated that geriatrician-led MDT management had practical significance in optimizing prescribing for older inpatients.

## Limitations

This study had several limitations. First, this study was a single-center retrospective study conducted in the Geriatric Center of West China Hospital of Sichuan University, with a relatively small clinical sample, and its generality in other parts of the country was unclear. Second, the research object of this study was only older patients in hospitals, and there was no relevant research in outpatients, communities or nursing homes. Thirdly, “Drug handover” is very important when patients switch healthcare settings. However few patients have their own family physician in China, which makes it difficult for patients to hand over medications after they are discharged. Therefore, we usually detail the patient’s medications at discharge in the discharge paperwork, and the patient and his or her caregiver are instructed on the use of the medications by the nurse practitioners. We also hope that in the future, more family physicians in China will be involved in the medication management of older patients with comorbidities. Therefore, a multicenter prospective randomized controlled trial should be carried out in the future to further confirm the effectiveness of geriatrician-led MDT intervention in medication management.

## Conclusion

This study found that implementing the geriatrician-led MDT mode during hospitalization had a significant effect on the management of medications, which could reduce the number of medications used and optimize prescription for older inpatients. Patients with polypharmacy were more likely to “description” after MDT management, while patients with COPD were more likely to be underprescription at home, which could be made up for after MDT management.

This study provides an important reference for the development of MDT modes in geriatric medicine in China. It also provided a feasibility strategy for the drug safety of older people. Since the implementation of the MDT model requires more healthcare providers to participate and increases medical costs, the promotion of the MDT management mode is challenging in China.

## Data Availability

The original contributions presented in the study are included in the article/supplementary material, further inquiries can be directed to the corresponding author.
